# Impact of Tumor Location and Variables Associated With Overall Survival in Patients With Colorectal Cancer: A Mayo Clinic Colon and Rectal Cancer Registry Study

**DOI:** 10.3389/fonc.2019.00076

**Published:** 2019-02-19

**Authors:** Cassia B. Wang, Faisal Shahjehan, Amit Merchea, Zhuo Li, Tanios S. Bekaii-Saab, Axel Grothey, Dorin T. Colibaseanu, Pashtoon M. Kasi

**Affiliations:** ^1^Division of Hematology and Medical Oncology, Mayo Clinic, Jacksonville, FL, United States; ^2^Colon and Rectal Surgery, Mayo Clinic, Jacksonville, FL, United States; ^3^Division of Biomedical Statistics and Informatics, Mayo Clinic, Jacksonville, FL, United States; ^4^Division of Medical Oncology, Mayo Clinic, Scottsdale, AZ, United States; ^5^Division of Medical Oncology, West Cancer Center, University of Tennessee, Memphis, TN, United States

**Keywords:** colorectal cancer, survival, tumor location, tumor heterogeneity, tumor sidedness

## Abstract

**Background:** Our study investigated the demographic characteristics of Mayo Clinic Colon and Rectal Cancer Registry patients and sought to associate tumor location with overall survival.

**Methods:** Using the cohort of patients seen at Mayo Clinic (Minnesota, Arizona, Florida) from 1972 to 2017, we obtained 26,908 colorectal adenocarcinoma patient records. Overall survival of patients with colorectal cancer was analyzed by sidedness (right vs. left) and location (right vs. left vs. rectum). Kaplan–Meier method was used to analyze all demographic and cancer variables available within the dataset to trace survival over a 35-year period. Subgroups within variables were compared to each other using log-rank test and considered significantly different at *P* < 0.05. Cox proportional hazards regression model was used to assess impact of tumor location while controlling for age, year of diagnosis, sex, tumor stage, and tumor grade. Cox regression models were used to evaluate the independent effect of cancer location on overall survival after adjusting for age, gender, year of diagnosis, and cancer stage. To further explore the potential interaction effect of cancer location with cancer stage and year of diagnosis, similar multivariable Cox model was fit stratified by cancer stage (1–3 vs. 4) and by year of diagnosis (<1980, 1980–2000, >2000).

**Results:** Overall survival differed significantly within all variables studied after Kaplan–Meier method analysis (*P* < 0.0001). Survival was higher in the left-side group when evaluated by tumor sidedness, and rectal cancer patients had the highest median survival (101.3 months). Right-sided cancer patients had the worst prognosis in both tumor location and sidedness analyses, with a median survival of 76.6 months. However, the stratified analysis showed that, the difference in survival between left- and right-sided cancer only existed in late cancer stage (stage 4) patients but not in early cancer stage; therefore, screening for CRC to pick cancer at an early stage can influence overall survival significantly.

**Conclusion:** These observations confirm some of the previous and recent studies on sidedness of colorectal cancer patients. Our analysis is novel in that it included patients of all stages rather than just stage IV metastatic patients. This initial study provides a platform to investigate more biologic and clinical factors associated with tumor location. Merging this dataset with other available datasets and previously conducted studies within the institution will provide a robust platform for multiple future studies and collaborations. Finally, appropriate screening can result in a decrease in incidence and mortality of CRC.

## Introduction

Colorectal cancer (CRC) is one of the leading causes of cancer in both men and women (1). Recent reports have suggested that tumor location and sidedness have some correlation to clinical outcomes (2, 3).

Differences between left and right colon cancers have been described previously in terms of tumor biology and presentation. While there are known genetic and pathologic variables, location may account for a fair proportion of heterogeneity in these tumors. One of the reasons postulated for the difference is the varied embryological origins of the left and right sides of the colon (the left colon originates from the hindgut and the right colon originates from the midgut) (4). For example, it has been reported recently that right-sided CRC do not derive benefit from anti-epidermal growth factor receptor antibodies compared to left-sided CRC (5). Furthermore, demographic trends exist in the separation of CRC based on location. There seems to be consensus among studies that right-sided CRC patients are diagnosed at a later age, and have an advanced stage (3, 6–8). Moreover, it has been observed that the proportion of women is higher among cases with right colon cancers compared to those with left colon cancers. There is a growing body of literature as to the degree tumor location predicts overall survival, but studies on large cohorts of patients are still needed (8).

The purpose of this study was to utilize our 3-site single institution (Minnesota, Arizona, Florida) cancer registry to assess differences in outcomes of right-sided and left-sided colon and rectal cancer patients seen between the years 1972 and 2017.

Our aim was to replicate and confirm existing findings in our own cohort of patients by taking a relatively straightforward and clinically relevant approach to the variables we included and using widely accepted statistical methods to predict overall survival based on tumor location. The study was also meant to assess the validity of the dataset by confirming outcomes of patients based on staging and grading of tumors. This would allow integration with other institutional datasets to help further answer questions related to CRC. We analyzed several ways of grouping patients with CRC to help provide comparison to other study cohorts already published (9).

## Materials and Methods

### Patient Selection

Institutional review board approval was obtained. Patient data were gathered from the Mayo Clinic Colon and Rectal Cancer Registry. These included patients evaluated at the Mayo Clinic campuses in Minnesota, Arizona, and Florida between the years 1972 and 2017. We obtained a total of 26,908 colorectal adenocarcinoma patient records.

Patients with colorectal adenocarcinomas were selected and grouped according to the location of their tumor. To allow for comparisons to previous and future studies, analysis on tumor location was studied in two ways. First, CRCs were divided into right- and left-sided tumor subgroups. In the second analysis, tumors were grouped into three subsets: right-sided colon, left-sided colon, and rectal cancers. For the analysis conducted on tumor sidedness (left or right), transverse colon cancers were excluded; and for tumor location analysis (left, right, or rectum), transverse colon, and rectosigmoid junction cancers were both excluded. These exclusions were meant to eliminate possible misclassified data in the database, which uses International Classification of Diseases system to help identify patients. The rectosigmoid junction may be classified as colon or rectal cancer, and embryologically, the transverse colon is composed arbitrarily approximately of two-thirds right colon and one-third left colon.

### Variables of Interest

Overall survival of patients with CRC was analyzed by sidedness (right vs. left) and location (right vs. left vs. rectum). Kaplan–Meier method analysis was conducted on all demographic and cancer variables to trace survival over a 35-year period. Subgroups within variables were compared to each other using log-rank test and considered significantly different at *P* < 0.05. Cox proportional hazards regression model analysis was used to assess the impact of tumor location while controlling for age, year of diagnosis, sex, tumor stage, and tumor grade.

As noted, tumor location was initially defined by sidedness (right vs. left) and location (right vs. left vs. rectum). We also included the continuous variables of age and year of diagnosis and the categorical variables of sex, race, tumor stage, and tumor grade. Race was separated into white, African American, Asian/Pacific Islander, and other. Cancers were classified into stage 0–IV by the TNM mixed staging, combining all data with the same numeric level (e.g., 1a, 1b, and 1c) into one group. Tumor grade was included as a separate variable since colorectal staging does not take grade into account. Treatment variables were not included for this analysis. Classification via staging was expected to control for variation in treatment in the different groups given the large sample size.

### Statistical Analysis

Summary statistics for continuous variables were reported as mean *(SD)* and median (range) while categorical variables were reported as frequency (%). The continuous variables of age and year of diagnosis were further grouped into five and three cohorts, respectively, for analysis with Kaplan–Meier method. Age grouping started at 50 years, which was the start age of CRC screening for average-risk individuals before the recent update in screening guidelines. Year of diagnosis was grouped into the following cohorts: prior to 1980, 1980 to 2000, and after 2000.

Overall survival since diagnosis at 5, 10, 15, 25, and 35 years were estimated using Kaplan–Meier method and compared between groups using log-rank test. Cox regression models were used to evaluate the independent effect of cancer location on overall survival after adjusting for age, gender, year of diagnosis, and cancer stage. Proportional hazard assumption was checked based on Schoen's method (10). Since proportional hazard assumption was violated for age and cancer stage, time-dependent coefficients were estimated for these two variables.

To further explore the potential interaction effect of cancer location with cancer stage and year of diagnosis, similar multivariable Cox model was fit stratified by cancer stage (1–3 vs. 4) and by year of diagnosis (<1980, 1980–2000, >2000)

All tests were two-sided with α level set at 0.05 for statistical significance. Analysis was performed using the SAS9.4 software package (SAS Institute Inc.).

## Results

A total of 26,908 patients were included in the study. The majority of patients were men (56.3%). The largest cohort by race was white (71.7%). The median age at the time of diagnosis was 67 years. The demographic characteristics are demonstrated in Table 1.

**Table 1:** Demographic and baseline variables.

	**Total (*N* = 26,908)**
**AGE AT DIAGNOSIS**	
N	26,907
Mean *(SD)*	65.3 (13.4)
Median	67.0
Q1, Q3	57.0, 75.0
Range	(3.0–129.0)
**AGE AT DIAGNOSIS**	
Missing	1
< 50	3,762 (14.0%)
51–60	5,250 (19.5%)
61–70	7,737 (28.8%)
71–80	6,970 (25.9%)
>80	3,188 (11.8%)
**YEAR OF DIAGNOSIS**	
N	26,908
Mean *(SD)*	1994.5 (13.1)
Median	1996.0
Q1, Q3	1983.0, 2006.0
Range	(1972.0–2017.0)
**YEAR OF DIAGNOSIS**	
< 1980	5,050 (18.8%)
1980–1999	10,932 (40.6%)
> = 2000	10,926 (40.6%)
**GENDER**	
Missing	5
Female	11,767 (43.7%)
Male	15,136 (56.3%)
**RACE**	
White	19,284 (71.7%)
Black	288 (1.1%)
Asian/Pacific islander	264 (1.0%)
Other	491 (1.8%)
Unknown	6,581 (24.5%)
**HOSPITAL SITE**	
Arizona	2,138 (7.9%)
Florida	2,559 (9.5%)
Rochester, Minnesota	22,177 (82.4%)
Multiple	34 (0.1%)

When divided by tumor side, 15,880 (67.7%) patients had tumors on the left side and 7,570 (32.3%) had tumors occurring on the right. When excluding rectosigmoid junction and adding the rectum category, there were 6,371 (29.0%) left-sided tumors and 8,051 (36.6%) rectal tumors. There was a fairly even distribution of tumors in each stage (excluding Stage 0). Cancer-related information is described in Table 2.

**Table 2:** Cancer-related information.

	**Total (*N* = 26,908)**
**CANCER SIDE (TRANSVERSE EXCLUDED)**	
Missing	3,458
Right	7,570 (32.3%)
Left	15,880 (67.7%)
**CANCER SIDE (TRANSVERSE AND RECTUM EXCLUDED)**	
Missing	11,509
Right	7,570 (49.2%)
Left	7,829 (50.8%)
**CANCER LOCATION (TRANSVERSE AND RECTOSIGMOID EXCLUDED)**	
Missing	4,916
Right	7,570 (34.4%)
Left	6,371 (29.0%)
Rectum	8,051 (36.6%)
**TUMOR SIZE**	
N	17,882
Mean *(SD)*	57.2 (100.1)
Median	43.5
Q1, Q3	30.0, 60.0
Range	(0.0–998.0)
**REGIONAL LYMPH NODE POSITIVE**	
N	16,514
Mean *(SD)*	1.6 (3.3)
Median	0.0
Q1, Q3	0.0, 2.0
Range	(0.0–63.0)
**REGIONAL LYMPH NODE EXAM**	
N	20,678
Mean *(SD)*	12.6 (12.5)
Median	10.0
Q1, Q3	3.0, 18.0
Range	(0.0–89.0)
**BEHAVIOR**	
2	769 (2.9%)
3	26,139 (97.1%)
**MIXED STAGE**	
Missing	5,920
0	754 (3.6%)
1	5,667 (27.0%)
2	4,754 (22.7%)
3	5,385 (25.7%)
4	4,428 (21.1%)
**FOLLOW UP YEARS SINCE CANCER DIAGNOSIS**	
N	26,864
Mean *(SD)*	7.5 (8.2)
Median	4.2
Q1, Q3	1.3, 11.2
Range	(0.0–44.5)

**Figure 1:**
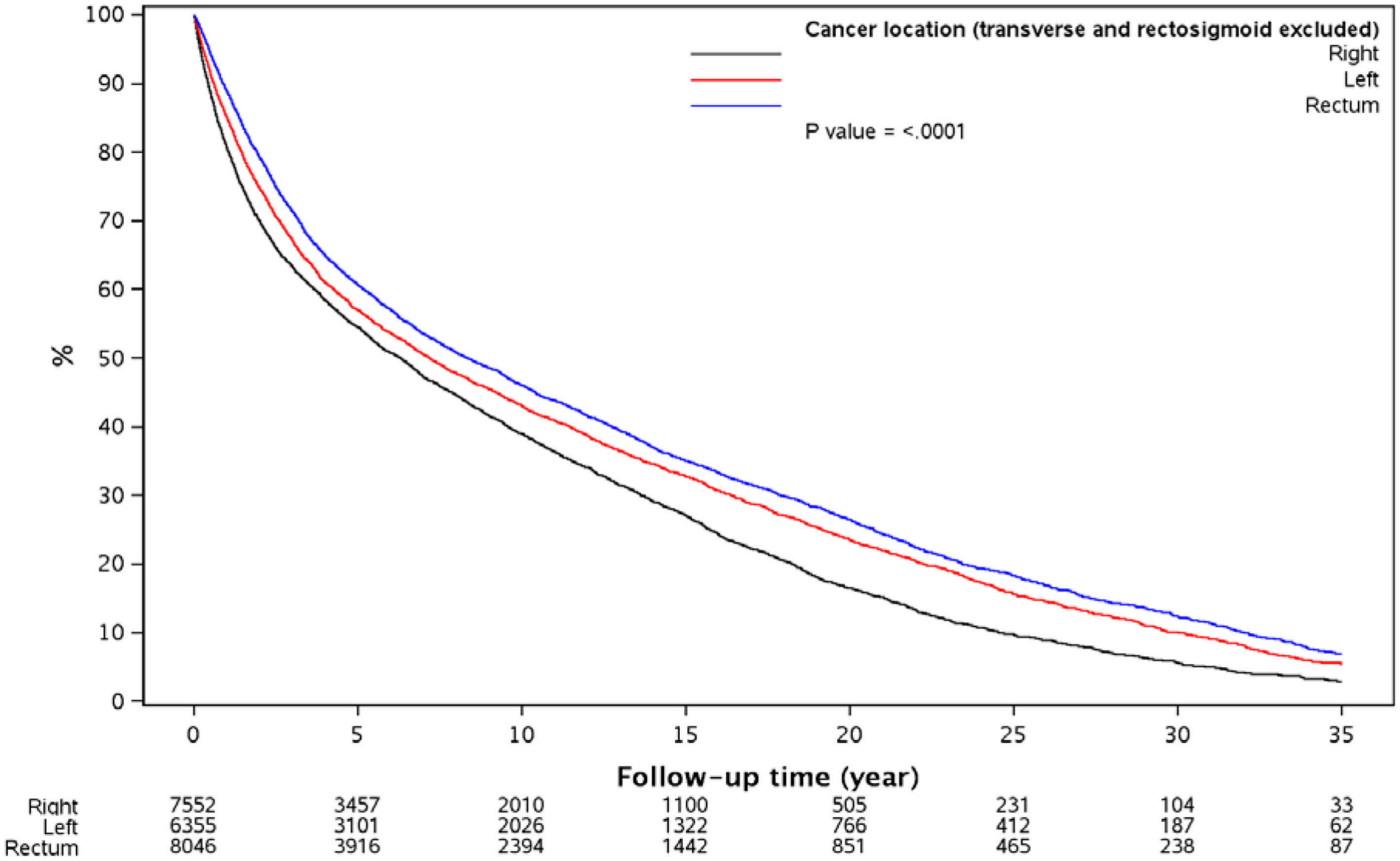
Overall survival since diagnosis by cancer location.

Kaplan–Meier method analysis, as demonstrated in Table 3, showed that all subgroups within each variable (sidedness or location) were significantly different from each other (*P* < 0.0001). Median survival for right-sided cancers was 76.6 vs. 93.0 months for left-sided cancers in analysis of tumor sidedness. Furthermore, in tumor location analysis, median survival for rectal cancer was the longest (101.3 months), followed by left-sided (87.5 months), and right-sided cancers (76.6 months). Survival difference between cancer locations was demonstrated in Figure 1.

**Table 3:** Kaplan–Meier estimates of overall survival since cancer diagnosis.

**Variable label**	**Formatted levels**	**Total number of patients**	**Total number of events**	**5-year survival (95%CI)**	**10-year survival (95%CI)**	**15-year survival (95%CI)**	**25-year survival (95%CI)**	**35-year survival (95%CI)**	**Median survival (months)**	***P*-value**
All patients	1	26,864	18,462	55.7% (55.0, 56.3)	41.1% (40.5, 41.7)	30.6% (29.9, 31.2)	14.4% (13.8, 14.9)	5.3% (4.8, 5.8)	81.1	
Age at diagnosis	< 50	3,759	1,744	60.3% (58.7, 62.1)	53.5% (51.7, 55.3)	50.1% (48.2, 52.0)	42.3% (40.2, 44.5)	30.7% (28.0, 33.7)	183.3	< 0.0001
Age at diagnosis	50–60	5,248	3,091	59.8% (58.4, 61.2)	50.7% (49.3, 52.3)	45.6% (44.1, 47.2)	30.2% (28.6, 32.0)	9.8% (8.3, 11.5)	129.2	
Age at diagnosis	60–70	7,724	5,478	58.7% (57.6, 59.9)	46.0% (44.8, 47.2)	35.3% (34.2, 36.6)	11.8% (10.9, 12.9)	1.0% (0.6, 1.6)	99.8	
Age at diagnosis	70–80	6,953	5,459	54.8% (53.6, 56.0)	35.0% (33.8, 36.2)	18.7% (17.7, 19.9)	1.6% (1.2, 2.1)	% (,)	74.0	
Age at diagnosis	>80	3,179	2,689	38.4% (36.7, 40.2)	15.4% (14.0, 16.8)	3.3% (2.6, 4.2)	% (,)	% (,)	40.3	
Gender	Female	11,750	7,936	56.9% (55.9, 57.8)	43.4% (42.4, 44.4)	33.4% (32.4, 34.4)	16.2% (15.3, 17.2)	6.2% (5.4, 7.1)	89.1	< 0.0001
Gender	Male	15,109	10,524	54.7% (53.9, 55.6)	39.3% (38.5, 40.2)	28.3% (27.5, 29.2)	12.9% (12.1, 13.7)	4.6% (4.0, 5.3)	76.4	
Race	White	19,261	11,858	60.2% (59.5, 61.0)	45.3% (44.5, 46.1)	34.4% (33.6, 35.2)	17.5% (16.7, 18.3)	6.8% (6.0, 7.6)	99.5	< 0.0001
Race	Black	288	152	61.5% (55.5, 68.0)	43.9% (37.4, 51.5)	32.9% (26.1, 41.5)	8.5% (3.6, 19.8)	5.6% (1.8, 18.1)	92.6	
Race	Asian/Pacific islander	264	77	66.0% (59.3, 73.5)	59.5% (52.0, 67.9)	51.7% (42.7, 62.6)	27.6% (10.9, 70.0)	% (,)	264.9	
Race	Other	489	364	44.5% (40.0, 49.5)	29.1% (24.9, 34.0)	20.5% (16.7, 25.2)	9.3% (6.5, 13.3)	3.4% (1.5, 8.0)	50.3	
Race	Unknown	6,562	6,011	43.5% (42.3, 44.7)	30.4% (29.3, 31.6)	21.4% (20.4, 22.4)	8.5% (7.8, 9.3)	2.8% (2.4, 3.4)	44.4	
Year of diagnosis	< 1980	5,036	4,827	43.5% (42.2, 44.9)	31.8% (30.6, 33.2)	24.3% (23.1, 25.5)	12.1% (11.2, 13.1)	4.6% (4.0, 5.2)	42.9	< 0.0001
Year of diagnosis	1980–1,999	10,908	9002	55.8% (54.9, 56.8)	41.7% (40.7, 42.6)	31.0% (30.1, 31.9)	14.1% (13.3, 14.8)	5.1% (4.3, 6.1)	82.8	
Year of diagnosis	> = 2000	10,920	4,633	62.1% (61.1, 63.1)	45.1% (43.8, 46.3)	32.3% (30.6, 34.0)	% (,)	% (,)	103.6	
Cancer side	Right	7,552	5,378	54.5% (53.4, 55.7)	39.0% (37.8, 40.2)	27.0% (25.9, 28.2)	9.7% (8.7, 10.7)	2.9% (2.2, 3.8)	76.6	< 0.0001
Cancer side	Left	15,857	10,551	58.5% (57.7, 59.3)	44.3% (43.4, 45.1)	33.7% (32.8, 34.5)	16.8% (16.0, 17.6)	6.2% (5.5, 6.9)	93.0	
Cancer location	Right	7,552	5,378	54.5% (53.4, 55.7)	39.0% (37.8, 40.2)	27.0% (25.9, 28.2)	9.7% (8.7, 10.7)	2.9% (2.2, 3.8)	76.6	< 0.0001
Cancer location	Left	6,355	4,513	57.0% (55.7, 58.3)	43.0% (41.7, 44.4)	32.8% (31.5, 34.1)	15.6% (14.5, 16.8)	5.4% (4.5, 6.5)	87.5	
Cancer location	Rectum	8,046	5,036	60.7% (59.5, 61.8)	46.1% (44.9, 47.3)	35.0% (33.8, 36.3)	18.3% (17.1, 19.6)	6.9% (5.9, 8.1)	101.3	
Mixed stage	0	753	376	83.6% (80.8, 86.4)	67.4% (63.6, 71.4)	49.4% (45.1, 54.0)	22.8% (18.8, 27.6)	10.9% (6.8, 17.6)	181.2	< 0.0001
Mixed stage	1	5,651	3,089	78.7% (77.6, 79.9)	60.0% (58.6, 61.5)	44.5% (42.9, 46.0)	20.0% (18.5, 21.7)	7.7% (6.0, 9.9)	158.8	
Mixed stage	2	4,750	2,806	70.8% (69.4, 72.2)	51.1% (49.6, 52.8)	36.2% (34.5, 37.9)	15.1% (13.5, 16.8)	5.4% (3.8, 7.6)	125.6	
Mixed stage	3	5,381	3,151	60.9% (59.5, 62.3)	44.6% (43.1, 46.1)	33.9% (32.3, 35.5)	16.6% (15.0, 18.3)	6.9% (5.2, 9.3)	97.0	
Mixed stage	4	4,419	3,724	15.4% (14.3, 16.6)	9.0% (8.0, 10.0)	7.3% (6.4, 8.3)	3.3% (2.4, 4.4)	1.5% (0.8, 2.9)	17.1	

**Figure 2:**
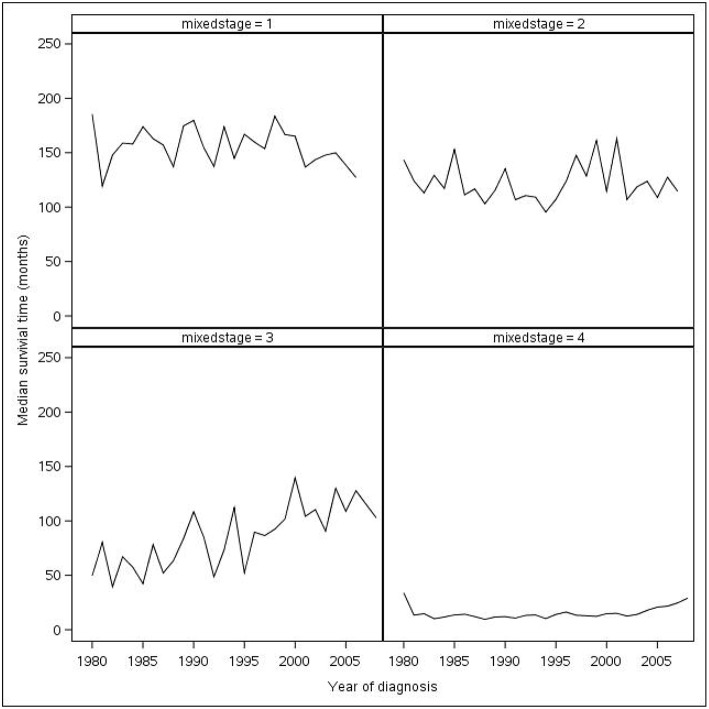
Median survival time vs. year of diagnosis based on stage (excluding years before 1980).

The year of diagnosis made a difference in survival, with those diagnosed in later years having longer median survival. Survival decreased as tumor stage increased as shown in Figure 2. The greatest drop in median survival was between stage III and stage IV CRC (97.0–17.1 months). Stage IV cancers had the worst prognosis with 15.4% 5-year survival. Women had a longer median survival time compared to men (89.1 vs. 76.4 months). Patients younger than 50 years of age survived the longest, with the median time being 183.3 months. Median survival vs. year of diagnosis based on cancer stage is shown in Figure 2. Multivariable Cox regression model shows that, after adjusting for age, gender, year of diagnosis, and cancer stage, left-sided cancer still showed a statistically significant survival advantage compared to right-sided cancer (HR = 0.90, 95%CI 0.86–0.94, *p* < 0.001) while rectum cancer patients showed no significant survival difference than right-sided cancer patients (Table 4). However, the stratified analysis showed that, the difference in survival between left- and right-sided cancer only existed in late cancer stage (stage 4) patients (Table 5) but not in early cancer stage (stage 1–3, Table 4). Similarly, the stratified analysis was done by diagnosis years, the difference between left and right sided cancer was statistically significant in the patients diagnosed between 1980 and 1999 (Table 6), but not the other two time periods, likely due to either smaller sample size, or shorter follow up.

**Table 4:** Multivariable model predicting overall mortality.

**Variable**	**Category**	**HR (95%CI)**	***P*-value**
Age at diagnosis	51–60 years old vs. < 50 up to 5 years FU	1.06 (0.96, 1.17)	0.2724
Age at diagnosis	61–70 years old vs. < 50 up to 5 years FU	1.31 (1.20, 1.44)	< 0.001
Age at diagnosis	71–80 years old vs. < 50 up to 5 years FU	1.88 (1.72, 2.06)	< 0.001
Age at diagnosis	>80 years old vs. < 50 up to 5 years FU	3.42 (3.10, 3.77)	< 0.001
Age at diagnosis	51–60 years old vs. < 50 after 5 years FU	1.97 (1.69, 2.29)	< 0.001
Age at diagnosis	61–70 years old vs. < 50 after 5 years FU	4.13 (3.58, 4.76)	< 0.001
Age at diagnosis	71–80 years old vs. < 50 after 5 years FU	9.00 (7.79, 10.40)	< 0.001
Age at diagnosis	>80 years old vs. < 50 after 5 years FU	18.55 (15.79, 21.80)	< 0.001
Gender	Male	1.21 (1.17, 1.26)	< 0.001
Year of diagnosis	1980–1999	1.46 (1.31, 1.63)	< 0.001
Year of diagnosis	> = 2000	1.12 (1.00, 1.26)	0.0439
Cancer location	Left vs. Right	0.90 (0.86, 0.94)	< 0.001
Cancer location	Rectum vs. Right	0.99 (0.95, 1.04)	0.786
Cancer stage	Stage1 vs. stage 0	1.14 (1.01, 1.27)	0.0322
Cancer stage	Stage2 vs. stage 0	1.37 (1.22, 1.54)	< 0.001
Cancer stage	Stage3 vs. stage 0	1.79 (1.60, 2.01)	< 0.001
Cancer stage	Stage4 vs. stage 0 up to 5 years FU	10.47 (9.28, 11.82)	< 0.001
Cancer stage	Stage4 vs. stage 0 after 5 years FU	3.29 (2.77, 3.93)	< 0.001

*Proportional hazard assumption was not met for age category and cancer stage, so these two factors were included in the model as time-dependent coefficients*.

**Table 5:** Multivariable Cox regression model predicting overall mortality, stratified by cancer stage.

**Variable**	**Category**	**HR (95%CI)**	***P* value**
**EARLY STAGE (1–3)**			
Age2before	51–60 years old vs. < 50 up to 5 years FU	1.04 (0.89, 1.21)	0.6433
Age3before	61–70 years old vs. < 50 up to 5 years FU	1.39 (1.21, 1.59)	< 0.001
Age4before	71–80 years old vs. < 50 up to 5 years FU	2.10 (1.84, 2.39)	< 0.001
Age5before	>80 years old vs. < 50 up to 5 years FU	4.08 (3.56, 4.68)	< 0.001
Age2after	51–60 years old vs. < 50 after 5 years FU	2.09 (1.77, 2.46)	< 0.001
Age3after	61–70 years old vs. < 50 after 5 years FU	4.50 (3.86, 5.25)	< 0.001
Age4after	71–80 years old vs. < 50 after 5 years FU	10.36 (8.87, 12.11)	< 0.001
Age5after	>80 years old vs. < 50 after 5 years FU	21.88 (18.43, 25.97)	< 0.001
Gender	Male	1.29 (1.23, 1.34)	< 0.001
Year diagnosis category	1980–1999	1.23 (1.07, 1.41)	0.003
Year diagnosis category	> = 2000	1.01 (0.87, 1.16)	0.9118
Cancer location	Left vs. Right	0.99 (0.93, 1.04)	0.6443
Cancer location	Rectum vs. Right	1.12 (1.06, 1.18)	< 0.001
Cancer stage	Stage1 vs. stage 0	1.14 (1.02, 1.28)	0.0258
Cancer stage	Stage2 vs. stage 0	1.40 (1.25, 1.58)	< 0.001
Cancer stage	Stage3 vs. stage 0	1.83 (1.63, 2.06)	< 0.001
**LATE STAGE (4)**			
Age2before	51–60 years old vs. < 50 up to 5 years FU	1.09 (0.95, 1.24)	0.2064
Age3before	61–70 years old vs. < 50 up to 5 years FU	1.25 (1.11, 1.41)	< 0.001
Age4before	71–80 years old vs. < 50 up to 5 years FU	1.68 (1.48, 1.91)	< 0.001
Age5before	>80 years old vs. < 50 up to 5 years FU	2.59 (2.23, 3.02)	< 0.001
Age2after	51–60 years old vs. < 50 after 5 years FU	1.30 (0.85, 1.99)	0.2201
Age3after	61–70 years old vs. < 50 after 5 years FU	2.32 (1.54, 3.48)	< 0.001
Age4after	71–80 years old vs. < 50 after 5 years FU	2.68 (1.69, 4.24)	< 0.001
Age5after	>80 years old vs. < 50 after 5 years FU	7.37 (2.23, 24.34)	0.0011
Gender	Male	1.05 (0.97, 1.13)	0.2266
Year diagnosis category	1980–2000	1.83 (1.53, 2.20)	< 0.001
Year diagnosis category	> = 2000	1.23 (1.02, 1.49)	0.0264
Cancer location	Left vs. Right	0.73 (0.67, 0.80)	< 0.001
Cancer location	Rectum vs. Right	0.76 (0.69, 0.83)	< 0.001

*Proportional hazard assumption was not met for age category, so age was included in the model as time-dependent coefficients*.

**Table 6:** Multivariable Cox regression model predicting overall mortality, stratified by diagnosis year.

**Variable**	**Category**	**HR (95%CI)**	***P*-value**
**YEAR OF DIAGNOSIS** < 1980^*^			
Age2before	51–60 years old vs. < 50 up to 5 years FU	0.92 (0.51, 1.66)	0.775
Age3before	61–70 years old vs. < 50 up to 5 years FU	1.02 (0.59, 1.79)	0.932
Age4before	71–80 years old vs. < 50 up to 5 years FU	1.05 (0.57, 1.93)	0.865
Age5before	>80 years old vs. < 50 up to 5 years FU	2.31 (1.09, 4.89)	0.029
Age2after	51–60 years old vs. < 50 after 5 years FU	2.87 (1.76, 4.69)	< 0.001
Age3after	61–70 years old vs. < 50 after 5 years FU	6.05 (3.64, 10.07)	< 0.001
Age4after	71–80 years old vs. < 50 after 5 years FU	10.24 (5.80, 18.10)	< 0.001
Age5after	>80 years old vs. < 50 after 5 years FU	21.40 (8.91, 51.40)	< 0.001
Gender	Male	1.14 (0.92, 1.42)	0.23
Cancer location	Left vs. Right	0.86 (0.65, 1.12)	0.259
Cancer location	Rectum vs. Right	0.90 (0.68, 1.19)	0.456
Cancer stage	Stage1 vs. stage 0	1.06 (0.62, 1.83)	0.826
Cancer stage	Stage2 vs. stage 0	1.20 (0.67, 2.13)	0.536
Cancer stage	Stage3 vs. stage 0	1.41 (0.81, 2.47)	0.225
Cancer stage	Stage4 vs. stage 0 up to 5 years FU	6.36 (3.66, 11.06)	< 0.001
Cancer stage	Stage4 vs. stage 0 after 5 years FU		
**YEAR OF DIAGNOSIS 1980–1999**			
Age2before	51–60 years old vs. < 50 up to 5 years FU	0.99 (0.87, 1.13)	0.883
Age3before	61–70 years old vs. < 50 up to 5 years FU	1.13 (1.00, 1.28)	0.045
Age4before	71–80 years old vs. < 50 up to 5 years FU	1.50 (1.34, 1.70)	< 0.001
Age5before	>80 years old vs. < 50 up to 5 years FU	2.63 (2.30, 3.00)	< 0.001
Age2after	51–60 years old vs. < 50 after 5 years FU	2.12 (1.77, 2.55)	< 0.001
Age3after	61–70 years old vs. < 50 after 5 years FU	5.10 (4.31, 6.04)	< 0.001
Age4after	71–80 years old vs. < 50 after 5 years FU	11.85 (9.98, 14.08)	< 0.001
Age5after	>80 years old vs. < 50 after 5 years FU	24.32 (19.98, 29.61)	< 0.001
Gender	Male	1.23 (1.17, 1.29)	< 0.001
Cancer location	Cancer location (transverse and rectosigmoid excluded) Left	0.88 (0.83, 0.94)	< 0.001
Cancer location	Cancer location (transverse and rectosigmoid excluded) Rectum	1.04 (0.98, 1.11)	0.156
Cancer stage	Stage1 vs. stage 0	1.03 (0.91, 1.18)	0.616
Cancer stage	Stage2 vs. stage 0	1.25 (1.09, 1.42)	0.001
Cancer stage	Stage3 vs. stage 0	1.65 (1.44, 1.88)	< 0.001
Cancer stage	Stage4 vs. stage 0 up to 5 years FU	7.51 (6.55, 8.61)	< 0.001
Cancer stage	Stage4 vs. stage 0 after 5 years FU		
**YEAR OF DIAGNOSIS** **> = 2000**			
Age2before	51–60 years old vs. < 50 up to 5 years FU	1.09 (0.93, 1.27)	0.271
Age3before	61–70 years old vs. < 50 up to 5 years FU	1.45 (1.26, 1.67)	< 0.001
Age4before	71–80 years old vs. < 50 up to 5 years FU	2.26 (1.96, 2.60)	< 0.001
Age5before	>80 years old vs. < 50 up to 5 years FU	4.06 (3.51, 4.69)	< 0.001
Age2after	51–60 years old vs. < 50 after 5 years FU	1.34 (0.95, 1.89)	0.091
Age3after	61–70 years old vs. < 50 after 5 years FU	2.33 (1.72, 3.17)	< 0.001
Age4after	71–80 years old vs. < 50 after 5 years FU	5.03 (3.74, 6.75)	< 0.001
Age5after	>80 years old vs. < 50 after 5 years FU	13.07 (9.61, 17.78)	< 0.001
Gender	Male	1.20 (1.12, 1.28)	< 0.001
Cancer location	Cancer location (transverse and rectosigmoid excluded) Left	0.92 (0.84, 1.00)	0.056
Cancer location	Cancer location (transverse and rectosigmoid excluded) Rectum	0.92 (0.85, 1.00)	0.045
Cancer stage	Stage1 vs. stage 0	1.61 (1.22, 2.14)	< 0.001
Cancer stage	Stage2 vs. stage 0	1.92 (1.45, 2.55)	< 0.001
Cancer stage	Stage3 vs. stage 0	2.43 (1.83, 3.21)	< 0.001
Cancer stage	Stage4 vs. stage 0 up to 5 years FU	12.83 (9.70, 16.97)	< 0.001
Cancer stage	Stage4 vs. stage 0 after 5 years FU		

*Proportional hazard assumption was not met for age category, so age was included in the model as time-dependent coefficients*.

* *Cancer stage information was missing for 90% patients diagnosed before 1980, the number of patients used for analysis of that period was 384*.

## Discussion

In this study, we primarily described the characteristics of the Mayo Clinic cohort of patients treated at all three sites from 1972 to 2017 (Mayo Clinic Colon and Rectal Cancer Registry). Additionally, we described the overarching trends in survival outcome by colorectal tumor sidedness and location within our cohort of patients.

A few assumptions were made regarding treatment (including surgery) and tumor staging. We did not consider the number of lymph nodes examined, which has shown to play a role in overall survival (7, 11, 12). However, including this would probably demand further research in differences in lymph node examination adequacy based on tumor location. With our large sample size within a single system enterprise cohort, we are confident that the correlations we found are significant. We were able to report on trends and outcomes that extended over 35 years which up to our knowledge is a unique observation. Although the number of possible causes of death increases with 35-year survival, we were still able to observe notable trends. Our selection of variables was kept to a minimum, allowing us to describe a broad overview of this dataset of patients before dividing into subset analyses and other biologic and therapy-related variables.

Previously studies have shown an association between CRC tumor sidedness and survival. Petrelli et al. did a systematic review and meta-analysis of 66 studies and demonstrated that left-sided CRC is associated with decreased mortality (HR: 0.82; 95% CI: 0.79–0.84) (13). Taieb et al. recently conducted a study on stage III colon cancer patients (*n* = 1,869) and their results showed that patients with right-sided tumors had poor overall survival (HR: 1.25; 95% CI: 1.02–1.54) compared to those with left-sided tumors (14). Another study revealed the association of left-sided tumor with better survival in patients with RAS wild-type metastatic CRC (15). Our study demonstrated the similar trends of survival thus corroborating the findings of previously published reports. It is also reported that young individuals are more likely to develop cancer of left colon and rectum than right colon (18).

It has been noticed that about 60–70% of patients who develop CRC-related symptoms are diagnosed at a late stage of cancer. Screening of CRC has revolutionized its management and serves as a marker of outcomes. Firstly, early diagnosis has a strong impact on survival since it shifts at least one of the two boundaries of survival time. Furthermore, screening is also effective in reducing colorectal cancer mortality, thus also the second boundary of survival time is moved. Finally, screening in CRC influences incidence, initially it increases and then decreases, thus changing the denominator of survival; this change is surely different for left and right cancers. Thus, appropriate screening can result in a decrease in incidence and mortality of CRC.

Characteristics regarding left colon, right colon, and rectal cancer have been discovered in previous studies and are summarized in Table 7. These include variations in biomarkers, such as caudal-type homeobox transcription factor 2 expression and C-reactive protein levels, where a significant association between higher C-reactive protein and right-sided colon cancer has been determined, and caudal-type homeobox transcription factor 2 negative expression has shown to have higher occurrence in metastatic right-sided colon cancer than in left-sided colon cancer (16, 19). Microsatellite instability and its impact on overall survival have also been studied, with notable differences between proximal and distal locations (20). Molecular differences within CRC are of interest as mutation and epigenetic data become more readily available through The Cancer Genome Atlas (21). Thorough analysis of these trends will have the potential to influence clinical practices and give rise to development of potential therapeutics specific to tumor location. Studying variations based on consensus molecular subtypes (CMS classification) would also be of value.

**Table 7:** Characteristics of right and left colorectal cancer.

**Characteristics**	**References**
• Older age at diagnosis for right-sided colon cancer	(3, 5, 7, 9, 16)
• Patients diagnosed with right-sided colon cancer more likely to be female • More poorly differentiated tumors in right-sided colon cancer	
• Comorbidities more common in patients with right-sided colon cancer	
• Higher stage at diagnosis for right-sided colon cancer	(7, 17)
• Number of lymph nodes examined is higher in right-sided colon cancer	
• Higher CA19–9 serum level in right-sided colon cancer	
• African Americans more likely to develop right-sided colon cancer	
• Left-sided colon cancer more commonly seen in patients with familial adenomatous polyposis, whereas right-sided colon cancer is associated more with MSI-High status	(16)
• Left-sided colon cancer are more often aneuploid and right-sided colon cancer more diploid	
• Right-sided colon cancer associated with higher C-reactive protein, which is associated with higher risk of morbidity	(7, 10, 11)
• CDX2-negative metastatic CRC more likely right-sided, and is also associated with worse overall survival	

Time trends are of importance and interest. It is difficult to mark out noteworthy clinical and biologic discoveries in survival curves. Therefore, we plan to examine survival rates for the year of diagnosis in subsequent studies. We may then be able to observe changes based on various factors, including, but not limited to cancer stage, age, and tumor location, and assess the differences in improvement. These trends will allow us to assess the true impact of modified clinical practices and treatments on variable-specific survival rates and add to our previous knowledge from this study (22).

Furthermore, we recognize the power of these types of clinical databases, which provide a large number of samples with detailed information. Data for one patient may be collected from various databases throughout Mayo Clinic and used for specific case studies. Pathology, radiology, and transplant databases are some useful databases to consider increasing collaboration among the departments and increasing our ability to answer clinical questions in a collaborative fashion.

The primary limitation of this study is its retrospective nature. We also did not study therapy-related variables. Furthermore, for some variables, such registries are not be of value; for example, we were not able to draw any conclusions from our analysis with race (17, 23). Ongoing expansion and merging of the database with other institutional datasets will help overcome these limitations and provide a robust platform for ongoing collaborations.

## Conclusion

Right-sided CRC is different from left-sided CRC in terms of survival for both early- and late-stage disease. This warrants further studies to better understand the tumor heterogeneity and underlying responsible biologic factors. Furthermore, tumor sidedness should be considered as an independent factor while choosing treatment strategies for managing CRC. Future collaborations to merge data from multiple institutions would be of value.

## Meeting

Abstract got published by American Society of Clinical Oncology (ASCO) on May 16, 2018 for 2018 ASCO Annual Meeting (June 1-5, 2018 at the McCormick Place Convention Center in Chicago, Illinois). Abstract #: e15651.

## Ethics Statement

The study was conducted under an IRB approved protocol. It is exempt from consent since it is a retrospective database study.

## Author Contributions

All authors listed have made a substantial, direct and intellectual contribution to the work, and approved it for publication.

### Conflict of Interest Statement

The authors declare that the research was conducted in the absence of any commercial or financial relationships that could be construed as a potential conflict of interest.
